# Biodistribution and residence time of adenovector serotype 5 in normal and immunodeficient mice and rats detected with bioluminescent imaging

**DOI:** 10.1038/s41598-017-03852-0

**Published:** 2017-06-15

**Authors:** Qiang Liu, Shuya Zhou, Changfa Fan, Weijin Huang, Qianqian Li, Susu Liu, Xi Wu, Baowen Li, Youchun Wang

**Affiliations:** 1 Division of HIV/AIDS and Sex-transmitted Virus Vaccines, National Institutes for Food and Drug Control, Beijing, 100050 China; 2 Division of Animal Model Research, Institute for Laboratory Animal Resources, National Institutes for Food and Drug Control, Beijing, 100050 China

## Abstract

As concerns increase about adenovirus type 5 (Ad5) being a safe gene transfer vector, it is important to evaluate its distribution, residence time, and possible toxicity in immunodeficient populations. To characterize the potential risk associated with different Ad5 vector delivery modes, we used immunocompetent and immunodeficient *Rag2* ^−/−^ animals to establish mouse and rat models that could be monitored with bioluminescent imaging following intramuscular or intravascular infection with an engineered replication-incompetent Ad5 virus carrying the firefly luciferase gene (Ad5-Fluc). The Ad5 vector was less well-tolerated by *Rag2* ^−/−^ animals than by wildtype ones, with delayed residence time, wider virus dissemination, less weight gain, and relatively severe pathological changes. In intravascularly Ad5-Fluc-infected *Rag2* ^−/−^ mice, systemic virus dissemination extended from the abdomen to the limbs and head on day 9 post-infection. Additionally, significant increases in plasma TNF-α and IFN-γ, which may be important factors in the heightened immunopathology in the liver and brain, were detected in the *Rag2* ^−/−^ mice 30 days after intravascular delivery. The Ad5 vector was better tolerated after intramuscular delivery than after intravascular delivery. Ad5-Fluc/*Rag2* ^−/−^ mice and rats can be used as reliable models of an immunodeficient population in which to evaluate the safety of Ad5-vectored vaccines or gene therapy products.

## Introduction

Adenovirus type 5 (Ad5) is widely used experimentally and clinically for gene delivery in oncology, cardioangiology, and regenerative medicine and is also used as a vaccine vector^1–3^. Replicating and non-replicating adenoviruses have been shown to be safe vectors in numerous clinical studies^4^. However, serious concerns regarding the safety of the Ad5 vector arose when a clinical trial was halted in response to its fatal adverse effects on an ornithine-transcarbamylase-deficient patient in 1999^5^. Therefore, a comprehensive understanding of the virological and biological features that define the infectivity and possible toxicity of Ad5 in different individuals is urgently required. Immunosuppression occurs in various types of patients, including those infected with human immunodeficiency virus (HIV), the recipients of organ transplantation, and those with hypogammaglobulinaemia^6, 7^. All of these patients are generally characterized by an increased and distinctive susceptibility to various types of pathogens, depending on the nature of the immune defect^8^. However, the behaviour, including biodistribution and residence time, of the Ad5 vector (Adv5) in the context of immunodeficiency is poorly understood.

We previously constructed a replication-competent vaccinia Tiantan strain infection model using an *in vivo* bioluminescent imaging (BLI) method and found that immunodeficient rats were more susceptible to vaccinia virus than Sprague Dawley (SD) rats and also displayed prolonged infections^9^. The BLI technology has the advantages of rapidity, simplicity, and quantitative precision, which allows the dissemination and clearance of viral vectors in intact and living animals to be carefully evaluated^10^. The aim of this study was to characterize the risk associated with different modes of Adv5 delivery, especially in immunodeficient hosts, and to identify the mechanisms underlying the resulting immunopathology. Therefore, we monitored the biodistribution and metabolism of replication-incompetent Ad5 expressing firefly luciferase (Ad5-Fluc) in real time in living mice and rats with or without immunodeficiency.

The immunodeficient *Rag2* ^−/−^ rats were generated previously to resemble immunodeficient human populations^9^. Here, both *Rag2* ^−/−^ mice and *Rag2* ^−/−^ rats were used for Ad5 vector safety evaluation. Like *Rag2* ^−/−^ rats, *Rag2* ^−/−^ mice are defective in the recombination machinery required for the development of both B and T cells, but these animals can produce NK cells, and no other innate immune cells are affected by the *Rag2* ^−/−^ mutation^11^. The immunodeficient *Rag2* ^−/−^ mice were more sensitive to Ad5 viral infection than the immunocompetent mice; when luminescent signals were detected with the BLI method, brain tissue sections showed pathological findings only in the *Rag2* ^−/−^ mice. Additionally, a tumour necrosis factor α (TNF-α)- and interferon γ (IFN-γ)-related cytokine storm occurred in the *Rag2* ^−/−^ mice that were infected intravascularly with Adv5, and the level of the cytokine storm correlated with the pathological changes in the liver and brain.

## Results

### Bioluminescent imaging of Adv5 infection in living mice

To test and verify the T and B cell development in homozygous *Rag2*-knockout mice, we used flow cytometry to identify various white blood cell populations in the peripheral blood. We detected significantly reduced numbers of T helper cells (CD3^+^/CD4^+^) in the *Rag2* ^−/−^ mice (0.18%) compared with the wildtype mice. The development of cytotoxic T cells (CD3^+^/CD8^+^) was also blocked in the *Rag2* ^−/−^ mice (0.09%). Additionally, we failed to detect CD3^*−*^/CD19^+^ B cells in the peripheral blood (0.35%) of the *Rag2* ^−/−^ mice. These results indicate that no mature T or B cells were produced after the *Rag2* gene was knocked out. However, we observed that the number of CD3^*−*^NK1.1^+^ natural killer (NK) cells increased in the *Rag2* ^−/−^ mice (77.3%) compared with the wildtype mice (11.1%) (Supplementary Fig. S1). This increase in NK cells may be a compensation mechanism to strengthen the immune system when mature T and B cells are abolished by a *Rag2* gene knockout.

**Figure 1:**
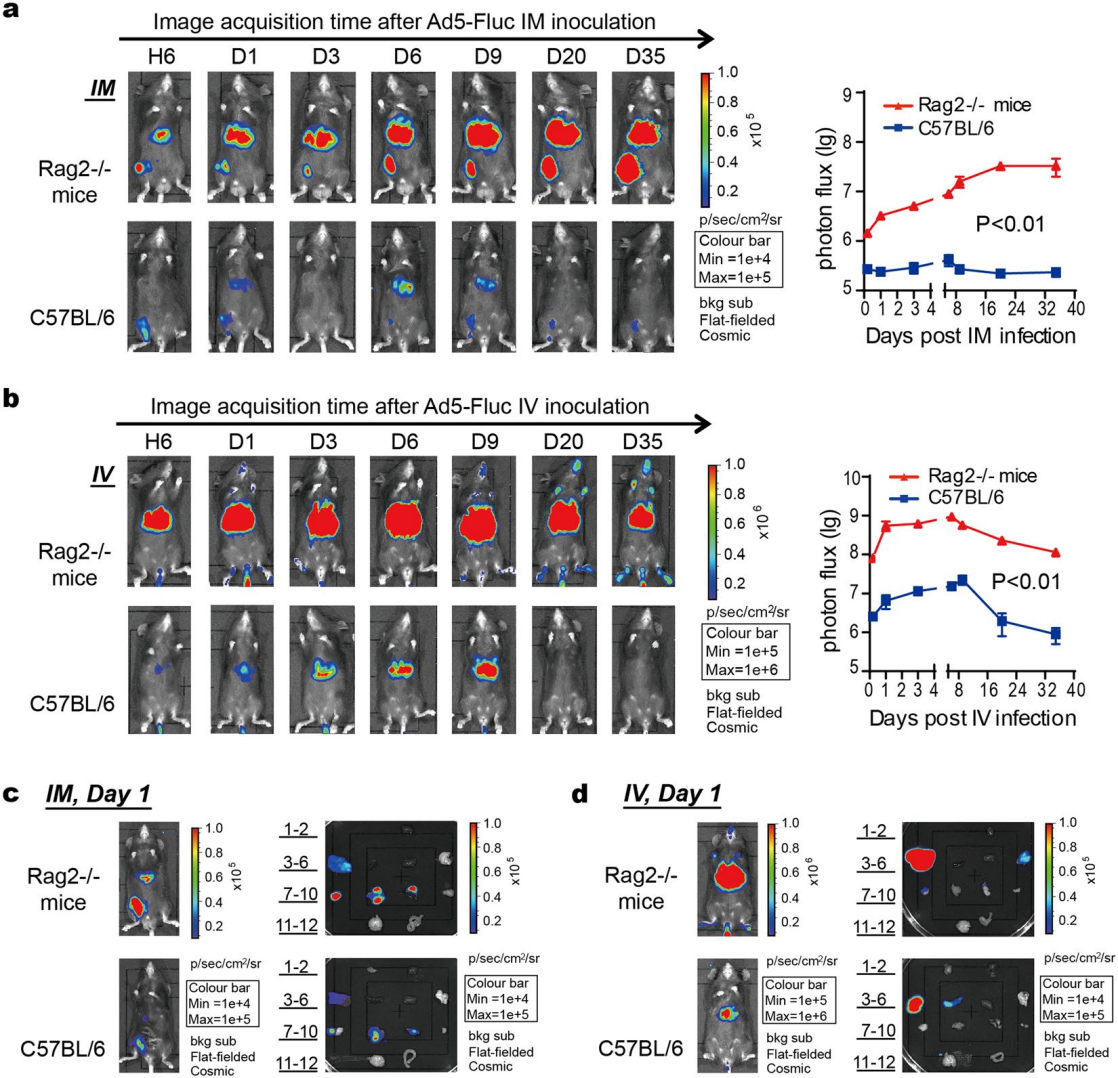
*In vivo* imaging of luciferase expression after inoculation of wildtype C57BL/6 and immunodeficient *Rag2* ^−/−^ mice with Ad5-Fluc. (**a**,**b**) Four-week-old *Rag2* ^−/−^ and C57BL/6 mice (n = 10/group) were inoculated with 5 × 10^10^ pfu/kg Adv5 by the IM (**a**) or IV route (**b**). Bioluminescent images of a representative mouse from each group are shown at 6 h, 1 day, 3 days, 6 days, 9 days, 20 days, and 35 days post-infection. Relative bioluminescence intensity is shown in pseudo-colour, with red and blue representing the strongest and weakest photon fluxes, respectively. Mean (±standard error of the mean) values for total flux at different time points are shown. The differences between subgroups were calculated with a paired *t*-test. (**c**,**d**) *Rag2* ^−/−^ and C57BL/6 mice inoculated with Ad5-Fluc by the IM route (**c**) or IV route (**d**) were imaged on day 1 post-inoculation. Different organs were dissected and tested for Fluc transgene expression with BLI: (1) thymus, (2) heart, (3) liver, (4) spleen, (5) kidney, (6) lung, (7) lymph node, (8) muscle, (9) skin, (10) ovary/testis, (11) brain, and (12) intestine.

Adv5 is commonly administered as a vaccine vector by the intramuscular (IM) route and as gene delivery vector in gene therapy by the intravascular (IV) route. To document the distribution and kinetics of Adv5 in both immunocompetent C57BL/6 and immunodeficient *Rag2* ^−/−^ mouse models, the animals were inoculated with 5 × 10^10^ plaque-forming units (pfu)/kg Adv5 by the IM or IV route. Because Ad5-Fluc expresses the bioluminescent luciferase protein, BLI was used to monitor the progress, sites, and degree of Adv5 expression in real time, based on the detected luciferase bioluminescence. *In vivo* luminescent signals were visible at the inoculation sites of both IM and IV injections and in the abdomen as early as 6 h after administration (Fig. 1a,b, left), suggesting that the bioluminescent signals generated by Ad5-Fluc are strong enough to show the distribution of Adv5, even in the very early stage of its dissemination. By day 9, the peak luminescence intensity and greatest systemic dissemination were observed, extending from the abdomen to the limbs and head in the *Rag2* ^−/−^ mice infected *via* the IV route. However, we observed a rapid damping of luciferase expression in the wildtype C57BL/6 mice, and Adv5 was almost cleared by day 20 post-inoculation, regardless of its route of delivery (IM or IV). Interestingly, the luciferase expression from Adv5-Fluc that was injected IM persisted and increased continuously in the *Rag2* ^−/−^ mice until the end of the experiment (day 35), but no systemic dissemination was observed (Fig. 1a, left). Without clearance of the Adv5-infected cells by T cells and B cells, the luciferase signal was significantly stronger and more persistent in the *Rag2* ^−/−^ mice, regardless of the inoculation route (P < 0.01; Fig. 1a,b, right). These data indicate that T cells and B cells are essential for the clearance of Adv5 administered by either the IM or IV route.

To determine the bioluminescence distributions *in vivo*, various organs or tissues from the Adv5-inoculated mice were dissected and analysed after the perfusion of luciferin on day 1 post-inoculation. In the IM inoculation group, bioluminescent signals were detected in the tissues around the inoculation site, including the lymph node, muscle, and skin, and in the liver, reflecting the dissemination kinetics from the muscle to the liver (Fig. 1c). Judging from the live imaging, the biodistribution of IV-inoculated Adv5 was wider in the *Rag2* ^−/−^ mice than in the C57BL/6 mice, especially in the limbs and mouth. However, the bioluminescent signals did not differ in any of the dissected organs other than the limbs and mouth, and luminescence was detected in the liver, spleen, and lung (Fig. 1d). This indicates that Adv5 inoculated into the blood by IV administration accumulates rapidly in the liver and then spreads to other organs.

### Bioluminescent imaging of Adv5 infection in Rag2^−/−^ and SD rats

**Figure 2:**
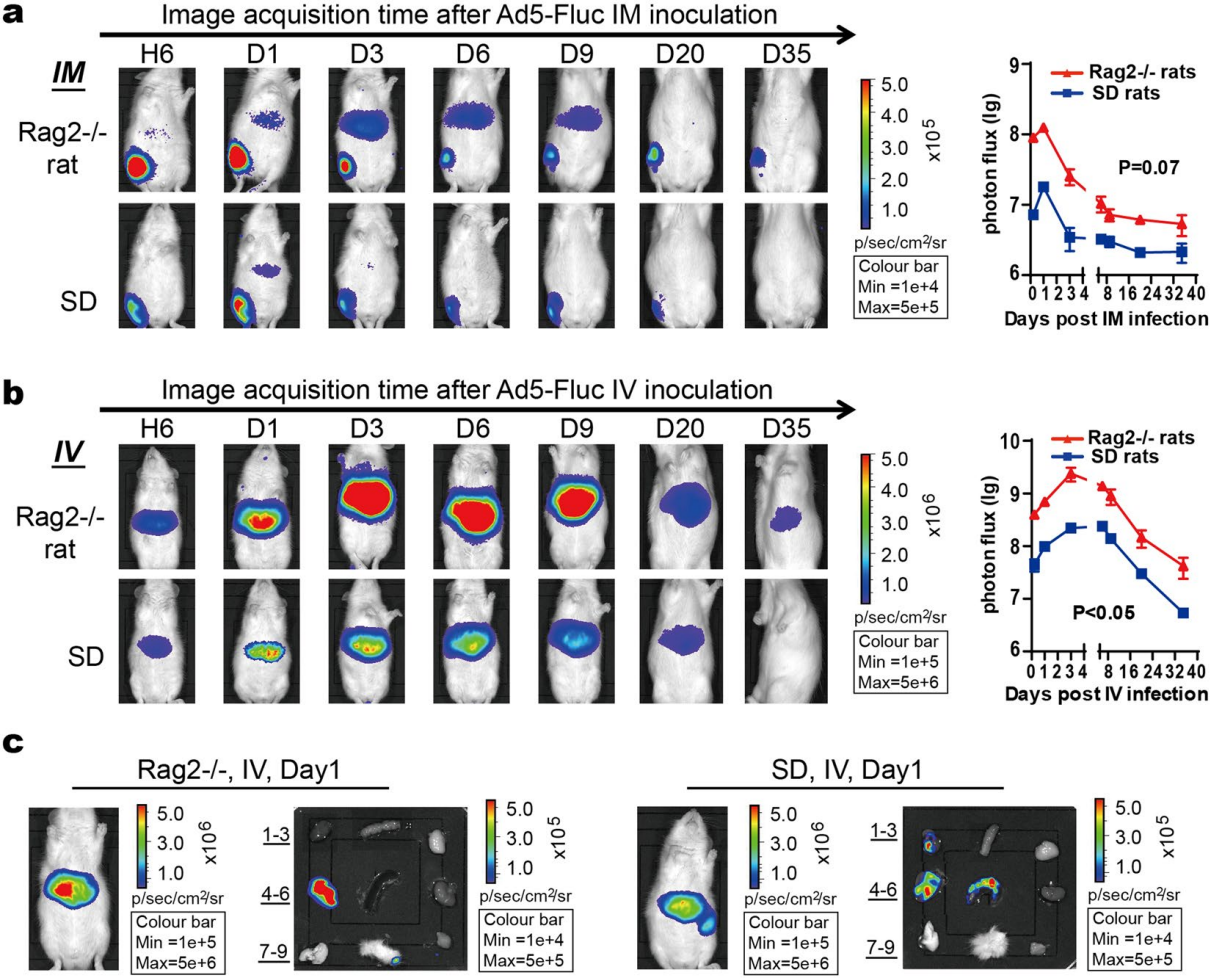
*In vivo* imaging of luciferase expression after inoculation of wildtype SD and immunodeficient *Rag2* ^−/−^ rats with Ad5-Fluc. (**a,b**) Four-week-old wildtype SD and immunodeficient *Rag2* ^−/−^ rats (n = 4/group) were inoculated with 5 × 10^10^ pfu/kg Ad5-Fluc *via* the IM (**a**) or IV (**b**) route. Bioluminescent images of a representative rat from each group at different time points following inoculation are shown. The relative bioluminescent intensity is shown in pseudo-colour, with red and blue representing the strongest and weakest photon fluxes, respectively. Mean (±standard error of the mean) values for the total flux at different time points are shown. The differences between subgroups were calculated using a paired *t*-test. (**c**) *Rag2* ^−/−^ and SD rats inoculated with Ad5-Fluc by the IV route were imaged on day 1 post-inoculation. Different organs were dissected and analysed for Fluc transgene expression with BLI: (1) heart, (2) intestine, (3) brain, (4) liver, (5) spleen, (6) kidney, (7) lung, (8) skin, and (9) muscle.

*Rag2* ^−/−^ rats, generated by applying the transcription activator-like effector nucleases (TALENs) technology, were observed to have no mature T or B cells; however, these animals had more NK cells than SD rats (Supplementary Fig. S2). After the administration of 5 × 10^10^ pfu/kg Adv5 *via* the IM or IV route, the initial local and systemic dissemination of Adv5 in rats were similar to those in mice. The *Rag2* ^−/−^ rats were more susceptible to Adv5 than the immunocompetent SD rats, displaying stronger luminescent signals and longer residence times (Fig. 2a,b, left). There were clear differences between the IV and IM delivery routes in terms of the biodistribution and residence time. After infection by the IM route, the Adv5 distribution was wider in the *Rag2* ^−/−^ rats than in the SD rats. However, the Adv5 distribution patterns were similar in the two rat strains after Adv5 administration by the IV route. Notably, the luciferase signal was significantly stronger and more persistent in the *Rag2* ^−/−^ rats infected by IV route than that in SD rats (P < 0.05; Fig. 2b, right). Notably, only small proportions of the *Rag2* ^−/−^ and SD rats infected by the IV route displayed infected spleens, but all of the rats displayed infected livers (Fig. 2c).

### Upregulated pro-inflammatory cytokine responses in Adv5-inoculated Rag2^−/−^ mice

**Figure 3:**
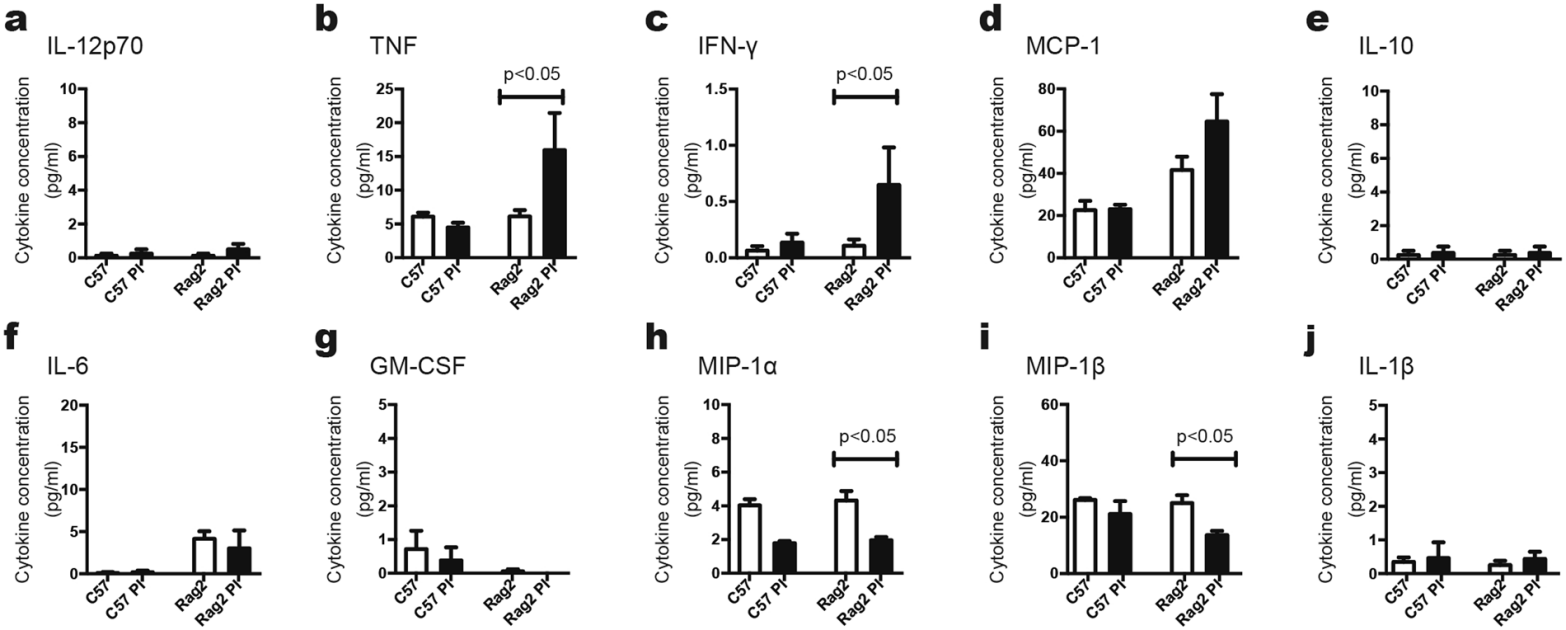
Levels of cytokines and chemokines in C57BL/6 and *Rag2* ^−/−^ mice pre- and post-Ad5-Fluc inoculation. Blood was sampled from C57BL/6 and *Rag2* ^−/−^ mice 1 day before and 30 days after Ad5-Fluc inoculation. Levels of IL-12p70 (**a**), TNF (**b**), IFN-γ (**c**), MCP-1 (**d**), IL-10 (**e**), IL-6 (**f**), GM-CSF (**g**), MIP-1α (**h**), MIP-1β (**i**), and IL-1β (**j**) were measured with the Cytometric Bead Array on a flow cytometer. Data are expressed as means ± SEM (n = 5/group). PI, post-inoculation.

Vaccine antigen clearance is predominantly dependent on the adaptive immune responses. Therefore, the striking difference between the Adv5 profiles of the wildtype and immunodeficient mice might be related to the magnitudes of their cellular immune responses. The cytokine dysregulation increases the risks associated with Adv5 inoculation in immunodeficient mice. We examined the magnitude of the cellular response induced in the immunodeficient *Rag2* ^−/−^ mice and the wildtype mice. Notably, the *Rag2* ^−/−^ mice showed no clearance of the Adv5-infected cells *in vivo*, at least at 35 days after IM or IV injection. Many pro-inflammatory cytokines in the blood, including the inflammatory cytokines interleukin (IL)-12p70, TNF-α, IFN-γ, IL-6, IL-10, granulocyte/macrophage colony-stimulating factor (GM-CSF), MIP-1α, MIP-1β, IL-1β, and chemokine monocyte chemoattractant protein 1 (MCP-1), were measured 1 day before and 30 days after inoculation. The cytokine levels showed little change in the C57BL/6 mice after Adv5 inoculation. Although the level of MIP-1α expression by C57BL/6 mice trended lower at 30 days post-inoculation, this difference did not reach statistical significance. Interestingly, the *Rag2* ^−/−^ mice had significantly lower expression levels of MIP-1α and MIP-1β than the C57BL/6 mice (P < 0.05; Fig. 3h,i), possibly due to the lack of mature T and B lymphocytes in these mice. As expected, the levels of TNF-α and IFN-γ were significantly higher in the post-Ad5-Fluc-inoculated *Rag2* ^−/−^ mice than in the pre-Ad5-Fluc-inoculated *Rag2* ^−/−^ mice (P < 0.05; Fig. 3b,c). However, although the MCP-1 levels tended to be higher in these mice after Adv5 inoculation, this difference did not reach statistical significance. When we compared the effects of IV and IM administration, although the levels of TNF-α and IFN-γ trend higher in the IV group than in the IM group, especially in the *Rag2* ^−/−^ mice, these differences are not statistically significant. Combined with the compensatory NK cell increase in the *Rag2* ^−/−^ mice that was detected with flow cytometry, these data suggest that the innate immune response was increased and prolonged in the *Rag2* ^−/−^ mice.

### Adv5 inoculation caused pathological changes in the brains of Rag2^−/−^, but not of C57BL/6 mice

**Figure 4:**
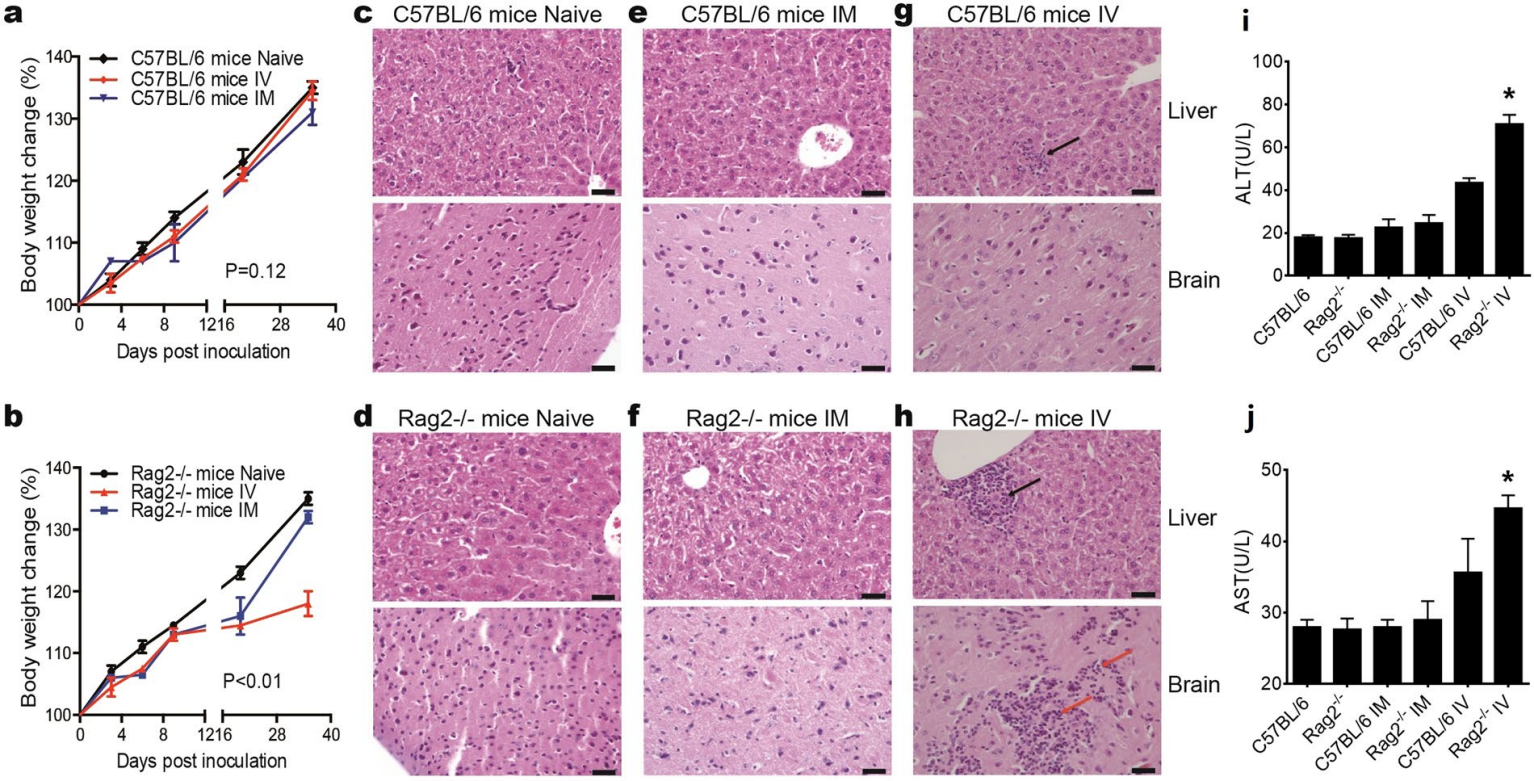
Body weight gain and pathological changes in mice inoculated with Ad5-Fluc. (**a**,**b**) Body weights of C57BL/6 mice (**a**) and *Rag2* ^−/−^ mice (**b**) inoculated with Ad5-Fluc *via* the IV or IM route were recorded (n = 5). (**c**,**h**) A histopathological analysis of each group, naïve C57BL/6 mice (**c**); naïve *Rag2* ^−/−^ mice (**d**); C57BL/6 mice inoculated *via* IM (**e**); *Rag2* ^−/−^ mice inoculated *via* IM (**f**); C57BL/6 mice inoculated *via* IV (**g**); and *Rag2* ^*−*/*−*^ mice inoculated *via* IV (**h**), was performed systematically on day 35 after inoculation with 5 × 10^10^ pfu/kg Ad5-Fluc. Paraffin-embedded tissue sections were stained with haematoxylin and eosin, and arrows indicate the lesion sites. Scale bar, 20 μm. Effects on the activities of serum (**i**) alanine aminotransferase (ALT) and (**j**) aspartate aminotransferase (AST). *Indicates that there is a significant (P < 0.05) difference between the treatment group and the control group.

To further investigate the potential risk of Adv5 inoculation in C57BL/6 and *Rag2* ^−/−^ mice, their body weights after Adv5 administration were monitored, and any pathological changes were analysed. No significant differences were detected between the body weight gains in the IV-infected, IM-infected, and naïve C57BL/6 mice (P = 0.12; Fig. 4a). However, the body weight gain in the IV-infected *Rag2* ^−/−^ mice was significantly lower than those in the other groups (P < 0.05; Fig. 4b). A systemic histopathological examination was performed in many tissues, including the brain, heart, liver, spleen, lung, kidney, intestine, thymus, muscle, and testis/ovary. No pathological changes were observed in the naïve C57BL/6 mice (Fig. 4c) or naïve *Rag2* ^−/−^ mice (Fig. 4d) or in the immunocompetent or immunodeficient mice inoculated with Adv5 *via* the IM route (Fig. 4e,f). Therefore, the IM route is relatively safe for use in the inoculation of Adv5-based vaccines. However, the IV-inoculated groups displayed inflammatory signs in the liver (Fig. 4g,h, upper). Moreover, glial nodules formed and oligodendrocytes increased in the brains of the immunodeficient *Rag2* ^−/−^ mice that were inoculated with Adv5 *via* the IV route. In contrast, these changes were not found in the brains of C57BL/6 mice infected *via* the IV route (Fig. 4g,h, lower). ALT is a cytosolic enzyme that mainly exists in the liver, while AST is primarily present in mitochondria and cytoplasm in the liver. Once hepatocytes are damaged, ALT and AST will leak into circulation and the levels of these enzymes will increase in serum. IV administration of Adv5 to the *Rag2* ^−/−^ mice increased serum alanine aminotransferase (ALT, Fig. 4i) and aspartate aminotransferase (AST, Fig. 4j) activities compared to that in control mice (P < 0.05). But there were no significant differences within the IM inoculated C57BL/6, *Rag2* ^−/−^ and naïve mice (Fig. 4i,j).

## Discussion

Here, we show that BLI is a valuable tool with which to visualize the distribution of Adv5 and the clearance of Adv5-infected cells after the virus administered by the IM or IV route to living mice and rats with or without immunodeficiency. This is a significant advance toward the development of Adv5-based therapies and vaccine improvement, and it extends our understanding of Adv5 immunopathology. In the context of preclinical drug safety evaluation, rats are believed to have a higher similarity to humans, providing predictable useful experimental data, and pharmaceutical companies use rats for a large proportion of their mandatory toxicity testing^12^. *Rag2* ^−/−^ rats, generated with the TALENs technology, were previously used to evaluate the toxicity and biodistribution of Adv5 in an immunodeficient population^9^. In this study, we found that Adv5 was more widely disseminated in the Rag2-deficient mice than in the Rag2-deficient rats, which suggests that these two rodent species differently predict the outcome in humans. However, in both species, the distributions and residence times in immunodeficient animals were different from those in immunocompetent ones, suggesting that Adv5 should be used with caution in various groups of immunosuppressed patients because considerably more serious adverse effects can be expected in these patients. Consistent with this, adenovirus is ubiquitous and causes generally mild, self-limiting infections in immunocompetent adults, but it can lead to a serious, often life-threatening illness in solid-organ transplant recipients and AIDS patients^13, 14^.

It was shown that after its IV administration, the Adv5 half-life was 2–5 min in normal mice and that more than 99% of Adv5 was cleared from the blood within 1 h. Most of the virus accumulated in the liver and was eliminated by Kupffer cells within 24 h^15^. In this study, the peak level of transgene expression was significantly higher and the kinetics of viral clearance was significantly slower in the T and B cell-deficient mice than in the wildtype mice after infection *via* either the IV or IM route. Therefore, we speculate that the T and B cells play important roles in the clearance of the Ad5 virus. Until now, the specific role of B cells during primary infection has been somewhat controversial. Although mice lacking both CD8^+^ T cells and B cells died after influenza infection, mice lacking CD4^+^ T cells and B cells survived^16^. However, Lee *et al*. showed that mice lacking B cells succumbed to influenza H1N1 (PR8) infection despite the infiltration of a larger number of CD8^+^ T cells^17^.

Conventional assays of host–pathogen interactions involving replication-competent and replication-defective human adenoviruses have been performed with PCR, Southern blotting, and immunohistochemical methods; however, these required large numbers of animals to be euthanized to obtain statistically meaningful data at multiple time points^18^. Here, to simplify this process of viral vector evaluation, we used BLI to monitor the dissemination of Adv5 and dynamically localize it in unexpected anatomical sites in the same animals by measuring the firefly luciferase expressed by Ad5-Fluc. We and others have also shown a significant correlation between photoemission and viral load *in vivo* and have demonstrated the many advantages of BLI, including the ability to image each animal repeatedly, which is both ethically and economically advantageous^19, 20^.

We found that Adv5 spread to the liver within 1 day of its IM administration. As reported previously, when Adv5 is delivered into muscle, it may infect the antigen-presenting cells that reside there, which then traffic it to the liver^21^. However, Adv5 can escape from the perivascular space into the systemic circulation *via* the vasa vasorum or through the lymphatics after its IM injection^22^. In contrast, after its IV administration, Adv5 rapidly binds to circulating platelets, causing their activation/aggregation and subsequent entrapment in the liver sinusoids. These virus–platelet aggregates are taken up by Kupffer cells and degraded^23^. We anticipate the future development of imaging strategies that detect not only the pathogen but also the cellular and tissue-wide reactions of the host. The study by Kadurugamuwa *et al*. that used multicolour bioluminescence to track bacterial spread and identify host neuronal injury was a key step towards the goal of monitoring both the host and the pathogen^24^.

Current evidence suggests that when Adv5 is used in gene therapy or vaccination, the innate immune responses are responsible for its dose-limiting toxicity and that the adaptive immune responses, which eliminate the infected cells, limit the duration of transgene expression^25^. We found that the *Rag2* ^−/−^ mice displayed significantly higher levels of the cytokines TNF-α and IFN-γ after Adv5 inoculation than the immunocompetent mice. Positive correlations between viral immunopathology and TNF-α or IFN-γ levels have also been reported in clinical cases and animal models of severe acute respiratory syndrome (SARS) and H5N1 infection^26, 27^. This release or synthesis of TNF, in turn, plays a major causative role in the pathological changes associated with IV adenovirus infusion as well as a role in the induction of the antiviral immune response^28, 29^. The IFN-γ produced by T and NK cells plays an important role in cell-mediated immunity against a broad spectrum of intracellular pathogens, and it also activates antigen presentation and primes TNF-α release by macrophages^30^. The inflammation associated with a cytokine storm has been shown to begin at a local site and to spread throughout the body *via* the systemic circulation^31^. Therefore, we speculate that the brain pathology in the immunodeficient mice was not a direct toxic effect of adenovector delivery, but more likely a secondary effect of inflammation and the immune response to the adenovector, because TNF-α and IFN-γ are considered the key cytokines that drive pro-inflammatory activity^32^.

Notably, hepatic injury was detected in both the *Rag2* ^−/−^ and wildtype C57BL/6 mice. Muruve showed that neutrophils acutely infiltrate the liver 1–6 h after the injection of Adv5. Immune effector cells are recruited to the sites of injury in response to chemokines, such as MCP-1, MIP-1α, MIP-1β, and RANTES. These signals directly correlate with the infiltration of inflammatory cells and the degree of hepatic injury^33^. Similar results were reported by Lieber, who reported a biphasic increase in serum TNF, with the first peak contributed by macrophages shortly after adenovirus administration and the second major peak contributed by lymphocytes after several days^34^. However, MIP-1α and MIP-1β decreased significantly after Adv5 infection in the *Rag2* ^−/−^ mice. MIP-1α and MIP-1β are produced by macrophages and activate human granulocytes, including neutrophils, eosinophils, and basophils, which may lead to acute neutrophilic inflammation^35^. Further research is required to determine whether or not the high-level expression of cytokines such as TNF-α and IFN-γ and the low levels of MIP-1α and MIP-1β in Adv5-infected mice are the causes or effects of the observed pathological changes. Moreover, the dynamics of cytokine expression during infection *in vivo* should also be investigated to detect any transient changes in cytokine levels. It is a pity that the data on cytokine response and histopathology were not conducted in rats, we believe that the conclusions will be more robust complementing with additional data in rats, and we will pay attention to this problem in the future study.

The current goal of vaccinologists and gene therapists is to create an Adv5 that avoids being detected by the immune system and has no deleterious side effects. We believe that the focus should be on two types of approaches. The first is dosage control. Some findings indicate that the hepatotropism of Adv5 is dose-limiting, and a certain threshold dose of injected Adv5 can saturate the capacity of Kupffer cells for adenovirus uptake, leading to the systemic dissemination of Adv5 particles. This then results in acute hepatocellular damage and the potential infection of other cell types that secrete pro-inflammatory cytokines, thus exacerbating the systemic toxicity of the vector^36^. The second approach involves modifying the method or route of delivery, using for instance, hydrodynamic injection, ultrasound-guided intramyocardial gene delivery, or other techniques to locally deliver low vector doses directly into the target organs, reducing the systemic dissemination of the vector, the inflammatory responses, and the immunotoxicity^37^.

Cytokine flare, reduced body weight gain, and pathological damage suggest that there is a possible risk involved in the use of Adv5 in gene therapy *via* the IV route, especially in immunodeficient populations. Although useful in preclinical studies, in general, the predictions from rodent studies have limited applicability to human outcomes. A single animal model can never recapitulate all of the aspects of human viral-vector-based gene therapies and vaccines because each model has its own advantages and disadvantages. Adenovirus-vectored vaccines have been constructed based on various different systems. In this study, recombinant Adv5 was generated using a Cre/loxP-based system^38^, which has been used in several vaccines, including an HIV vaccine^39^, and in gene therapy^40^. Notably, the promoter and overall design of the Ad5 expression cassette has a major impact on Adv5 toxicity and distribution *in vivo*. Therefore, the safety of recombinant Ad5 products generated with other construction systems must be evaluated in future studies. Additionally, the relative data should be obtained from older animals because gene therapy is predominantly administered to adults with degenerated tissues/cells.

In summary, in this study, we tested the residence time, biodistribution, and possible toxicity of Adv5 in both immunodeficient and immunocompetent mice and rats, with doses equivalent to the higher end of a reasonable range in humans. We found that the ideal route of administration was IM, by which even immunodeficient animals were successfully and safely treated with a high dose of the vaccine. Adverse effects were observed when the animals were treated by the IV route, which has been used in previous gene therapy applications. Therefore, in accord with previous reports, we believe that Ad5 vectors are generally safe for vaccine and gene therapy applications. However, future research should focus on any adverse effects in recipients with congenital immunodeficient backgrounds or virus-induced immunodeficiency. Finally, visualizing Ad5-Fluc infection in immunocompetent and immunodeficient mice and rats is an additional or alternative way to study adenoviral infection because it is convenient, sensitive, and requires the use of fewer animals.

## Materials and Methods

### Construction of Ad5-Fluc virus

Using the firefly luciferase (*Fluc*) gene derived from pLUCF (kindly provided by John T. Schiller, National Cancer Institute, Bethesda, MD, USA), a replication-incompetent Ad5-Fluc was constructed by deleting the E1 and E3 viral genes using the Cre/loxP-based system^38^. Briefly, PCR was used to construct a recombinant shuttle plasmid, pDC316-Fluc. HEK293 cells were co-transfected with an expression plasmid containing structural genes (pBHGlox-E1,3Cre) and the reporter plasmid for Ad5-Fluc construction using Lipofectamine 2000 (Invitrogen, Carlsbad, CA, USA). The recombinant adenovirus containing the Fluc gene (Ad5-Fluc) was acquired by Cre-lox recombination in HEK293 cells.

### Flow cytometry

To detect T, B, and NK cells in four-week old C57BL/6 and *Rag2* ^−/−^ mice, a multicolour cytometric analysis was performed using a BD FACSCalibur flow cytometer (Becton Dickinson, Franklin Lakes, NJ, USA) according to the manufacturer’s protocol. Peripheral blood was collected from the inner canthus of the mice. The blood was depleted of erythrocytes with lysing solution (BD PharMingen, San Diego, CA, USA). White blood cells were examined by doubly staining them with antibodies, including anti-CD3E–fluorescein isothiocyanate (FITC; cat: #553061), anti-CD4–phycoerythrin (PE; cat: #553652), anti-CD8–allophycocyanin (APC; cat: #553035), anti-CD19–APC (cat: #550992), and anti-NK1.1–PE (cat: #557391). All antibodies were purchased from BD PharMingen.

### Animal experiments

The mice and rats used in this study were housed and handled strictly in accordance with the guidelines set by the Association for the Assessment and Accreditation of Laboratory Animal Care (AAALAC; Frederick, MD, USA). The study protocol was approved by the Animal Care and Use Committee of the National Institute for Food and Drug Control (NIFDC; Beijing, China). Four-week-old *Rag2* ^−/−^ mice and *Rag2* ^−/−^ rats, wildtype C57BL/6 mice, and SD rats were obtained from the Institute for Laboratory Animal Resources, NIFDC. Eight groups each of mice (ten per group) and rats (four per group) received a single dose of Ad5-Fluc at 5 × 10^10^ pfu/kg (suspended in 100 μl of PBS) by IM inoculation or 5 × 10^10^ pfu/kg (suspended in 200 μl of PBS) by IV (tail vein) injection. This dose is equivalent to the higher end of a reasonable range in humans^41, 42^. The mice and rats were examined with BLI at 6 h, 1 day, 3 days, 6 days, 9 days, 20 days, and 35 days post-infection. Ten additional *Rag2* ^−/−^ and normal animals mice were used as negative controls. To test the immunopathology of Ad5-Fluc in mice, their body weights were monitored and recorded. The mice and rats were humanely euthanized with isoflurane to analyse the biodistribution of Ad5-Fluc in their tissues on day 1 post-infection.

### BLI analysis

Bioluminescent imaging was performed with the IVIS Lumina Series III Imaging System (PerkinElmer, Baltimore, MD, USA), as described previously^43^. In brief, the mice and rats were anesthetized with successive intraperitoneal injections of pelltobarbitalum natricum (240 mg/kg body weight) and the substrate d-luciferin (50 mg/kg body weight; Xenogen-Caliper Corp., Alameda, CA, USA). The mice and rats were imaged 10 min later. To image individual tissues, mice and rats were dissected rapidly, and the bioluminescence was quantified by using the IVIS system to analyse the tissues *ex vivo* within 3 min of dissection. Luminescence was acquired with an exposure time of 60 s, and regions of interest (ROIs) were analysed with the Living Image software (Caliper Life Sciences, Baltimore, MD, USA). The relative intensities of the emitted light are presented as pseudo-coloured images, with colours ranging from red (most intense) to blue (least intense). The signals emitted from different ROIs in the body were measured and are presented as the total flux in photons/s. All data are presented as mean values ± SEM.

### Cytokine quantification

The plasma was separated from EDTA-treated blood samples by centrifugation (2000 × *g* for 10 min) at 4 °C. Cytokines, including IL-12p70, TNF, IFN-γ, IL-6, IL-10, GM-CSF, MIP-1α, MIP-1β, IL-1β, and MCP-1, were quantified with the Cytometric Bead Array (BD Biosciences) on a FACSCalibur cytometer (BD Biosciences); this method has been used previously to analyze the immunopathology of different viral infections in humans^44–46^. Individual cytokine concentrations (pg/ml) were computed with standard reference curves in the CellQuest™ (BD Biosciences) and FCAP Array software, according to the manufacturer’s protocols. The inflammatory cytokines were measured simultaneously with different capture beads. Each capture bead in the array had a unique fluorescence and was coated with a capture antibody specific for a single analyte. A combination of different beads was mixed with a sample or standard with a mixture of detection antibodies that were conjugated to the reporter molecule (PE). After incubation and subsequent washing, the samples were analysed on a flow cytometer.

### Histopathology and biochemical analysis

Tissues, including the heart, lung, liver, spleen, kidney, intestine, thymus, skin, muscle, and brain, were dissected from the mice on day 35 post-infection, fixed in 10% formalin, and embedded in paraffin. Paraffin sections (2 μm thick) were stained with haematoxylin and eosin, and pathological changes were observed under a light microscope. Plasma alanine aminotransferase (ALT) and aspartate aminotransferase (AST) activities were measured using an enzyme-linked immune sorbent assay (ELISA) kit (China), according to the manufacturer’s instructions.

### Statistical analysis

All graphs were generated with Prism 5.0 software (GraphPad, San Diego, CA, USA). The differences in the various cytokine levels in the pre- and post-infection subgroups and the differences in the bioluminescent intensities in the *Rag2* ^−/−^ and normal subgroups were calculated with paired *t*-tests. P values < 0.05 were considered statistically significant.

## Electronic supplementary material

Supplementary figures

## References

[1] Schenk-Braat EA, van Mierlo MM, Wagemaker G, Bangma CH, Kaptein LC (2007). An inventory of shedding data from clinical gene therapy trials. J Gene Med.

[2] Reid T, Warren R, Kirn D (2002). Intravascular adenoviral agents in cancer patients: lessons from clinical trials. Cancer Gene Ther.

[3] Hammer SM (2013). Efficacy trial of a DNA/rAd5 HIV-1 preventive vaccine. N Engl J Med.

[4] Majhen D (2014). Adenovirus-based vaccines for fighting infectious diseases and cancer: progress in the field. Hum Gene Ther.

[5] Raper SE (2003). Fatal systemic inflammatory response syndrome in a ornithine transcarbamylase deficient patient following adenoviral gene transfer. Mol Genet Metab.

[6] Teich N (2011). Vaccination coverage in immunosuppressed patients: results of a regional health services research study. Dtsch Arztebl Int.

[7] Fraser C (2009). Pandemic potential of a strain of influenza A (H1N1): early findings. Science..

[8] George MP (2014). Infections in the immunosuppressed host. Ann Am Thorac Soc..

[9] Liu Q (2015). Bioluminescent imaging of vaccinia virus infection in immunocompetent and immunodeficient rats as a model for human smallpox. Sci Rep.

[10] Pan W (2013). Visualizing influenza virus infection in living mice. Nat Commun..

[11] Shinkai Y (1992). RAG-2-deficient mice lack mature lymphocytes owing to inability to initiate V(D)J rearrangement. Cell..

[12] Abbott A (2004). Laboratory animals: the Renaissance rat. Nature..

[13] Ying B (2014). Ganciclovir inhibits human adenovirus replication and pathogenicity in permissive immunosuppressed Syrian hamsters. Antimicrob Agents Chemother.

[14] Toth K (2008). Hexadecyloxypropyl-cidofovir, CMX001, prevents adenovirus-induced mortality in a permissive, immunosuppressed animal model. Proc Natl Acad Sci USA.

[15] Alemany R, Suzuki K, Curiel DT (2000). Blood clearance rates of adenovirus type 5 in mice. J Gen Virol.

[16] Mozdzanowska K, Maiese K, Gerhard W (2000). Th cell-deficient mice control influenza virus infection more effectively than Th- and B cell-deficient mice: evidence for a Th-independent contribution by B cells to virus clearance. J Immunol..

[17] Lee BO (2005). CD4 T cell-independent antibody response promotes resolution of primary influenza infection and helps to prevent reinfection. J Immunol..

[18] Shayakhmetov DM, Li ZY, Ni S, Lieber A (2004). Analysis of Adenovirus Sequestration in the Liver, Transduction of Hepatic Cells, and Innate Toxicity after Injection of Fiber-Modified Vectors. J Virol..

[19] Zhou S (2016). A safe and sensitive enterovirus A71 infection model based on human SCARB2 knock-in mice. Vaccine..

[20] Guse K (2007). Luciferase imaging for evaluation of oncolytic adenovirus replication *in vivo*. Gene Ther.

[21] Tatsis N (2007). Adenoviral vectors persist *in vivo* and maintain activated CD8+ T cells: implications for their use as vaccines. Blood..

[22] Hiltunen MO (2000). Biodistribution of adenoviral vector to nontarget tissues after local *in vivo* gene transfer to arterial wall using intravascular and periadventitial gene delivery methods. FASEB J.

[23] Stone D (2007). Adenovirus-platelet interaction in blood causes virus sequestration to the reticuloendothelial system of the liver. J Virol..

[24] Kadurugamuwa JL (2005). Reduction of astrogliosis by early treatment of pneumococcal meningitis measured by simultaneous imaging, *in vivo*, of the pathogen and host response. Infect Immun..

[25] Gregory SM, Nazir SA, Metcalf JP (2011). Implications of the innate immune response to adenovirus and adenoviral vectors. Future Virol..

[26] Cameron MJ (2007). Interferon-mediated immunopathological events are associated with atypical innate and adaptive immune responses in patients with severe acute respiratory syndrome. J Virol..

[27] de Jong MD (2006). Fatal outcome of human influenza A (H5N1) is associated with high viral load and hypercytokinemia. Nat Med.

[28] Shayakhmetov DM, Li ZY, Ni S, Lieber A (2004). Analysis of adenovirus sequestration in the liver, transduction of hepatic cells, and innate toxicity after injection of fiber-modified vectors. J Virol..

[29] Lozier JN (2002). Toxicity of a first-generation adenoviral vector in rhesus macaques. Hum Gene Ther.

[30] Engler H (2004). Acute hepatotoxicity of oncolytic adenoviruses in mouse models is associated with expression of wild-type E1a and induction of TNF-alpha. Virology..

[31] Rubenfeld GD (2005). Incidence and outcomes of acute lung injury. N Engl J Med.

[32] Xu L (2004). Cutting edge: pulmonary immunopathology mediated by antigen-specific expression of TNF-alpha by antiviral CD8+ T cells. J Immunol..

[33] Muruve DA, Barnes MJ, Stillman IE, Libermann TA (1999). Adenoviral gene therapy leads to rapid induction of multiple chemokines and acute neutrophil-dependent hepatic injury *in vivo*. Hum Gene Ther.

[34] Lieber A (1997). The role of Kupffer cell activation and viral gene expression in early liver toxicity after infusion of recombinant adenovirus vectors. J Virol..

[35] Maier R (2008). Multiplex bead analysis of vitreous and serum concentrations of inflammatory and proangiogenic factors in diabetic patients. Mol Vis..

[36] Liu Q (2003). The role of capsid-endothelial interactions in the innate immune response to adenovirus vectors. Hum Gene Ther.

[37] Brunetti-Pierri N (2007). Pseudo-hydrodynamic delivery of helper-dependent adenoviral vectors into non-human primates for liver-directed gene therapy. Mol Ther..

[38] Ng P (1999). A high-efficiency Cre/loxP-based system for construction of adenoviral vectors. Hum Gene Ther.

[39] Yu S (2008). Potent specific immune responses induced by prime-boost-boost strategies based on DNA, adenovirus, and Sendai virus vectors expressing gag gene of Chinese HIV-1 subtype B. Vaccine..

[40] Maeda M (2006). Targeted gene therapy toward astrocytoma using a Cre/loxP-based adenovirus system. Brain Res..

[41] Nicholson, O. *et al*. Safety and Immunogenicity of the MRKAd5 gag HIV Type 1 Vaccine in a Worldwide Phase 1 Study of Healthy Adults. *AIDS Res Hum Retroviruses* (2010). 10.1089/aid.2010.0151 PMC3422055 20854108

[42] Lang FF (2003). Phase I trial of adenovirus-mediated p53 gene therapy for recurrent glioma: biological and clinical results. J Clin Oncol.

[43] Zaitseva M (2009). Application of bioluminescence imaging to the prediction of lethality in vaccinia virus-infected mice. J Virol..

[44] Sun P (2006). CD40 ligand enhances dengue viral infection of dendritic cells: a possible mechanism for T cell-mediated immunopathology. J Immunol..

[45] Shrestha B (2006). Gamma interferon plays a crucial early antiviral role in protection against West Nile virus infection. J Virol..

[46] Yu XL (2008). Measles virus infection in adults induces production of IL-10 and is associated with increased CD4+ CD25+ regulatory T cells. J Immunol..

