# Supplementary material for: Biodistribution and residence time of adenovector serotype 5 in normal and immunodeficient mice and rats detected with bioluminescent imaging
Source: Sci Rep. 2017 Jun 15;7:3597. doi: 10.1038/s41598-017-03852-0 (PMC5472566; doi:10.1038/s41598-017-03852-0)
Supplement: Supplementary file 1 — Supplementary figures [file 41598_2017_3852_MOESM1_ESM.pdf]

Biodistribution and residence time of adenovector serotype 5 in normal and immunodeficient mice and rats detected with bioluminescent imaging

Qiang Liu[1]*, Shuya Zhou[2]*, Changfa Fan[2], Weijin Huang[1], Qianqian Li[1], Susu Liu[2], Xi Wu[2], Baowen Li[2], & Youchun Wang[1]

[1] Division of HIV/AIDS and Sex-transmitted Virus Vaccines, National Institutes for Food and Drug Control, Beijing, 100050, China

[2]Division of Animal Model Research, Institute for Laboratory Animal Resources, National Institutes for Food and Drug Control, Beijing, 100050, China

*These authors contributed equally to this work.

Correspondence: Youchun Wang, MD, PhD, National Institutes for Food and Drug Control, No. 2 Tiantanxili, Beijing 100050, China. Tel.: +86 10 67095921; Fax: +86 10 67095795; E-mail: wangyc@nifdc.org.cn

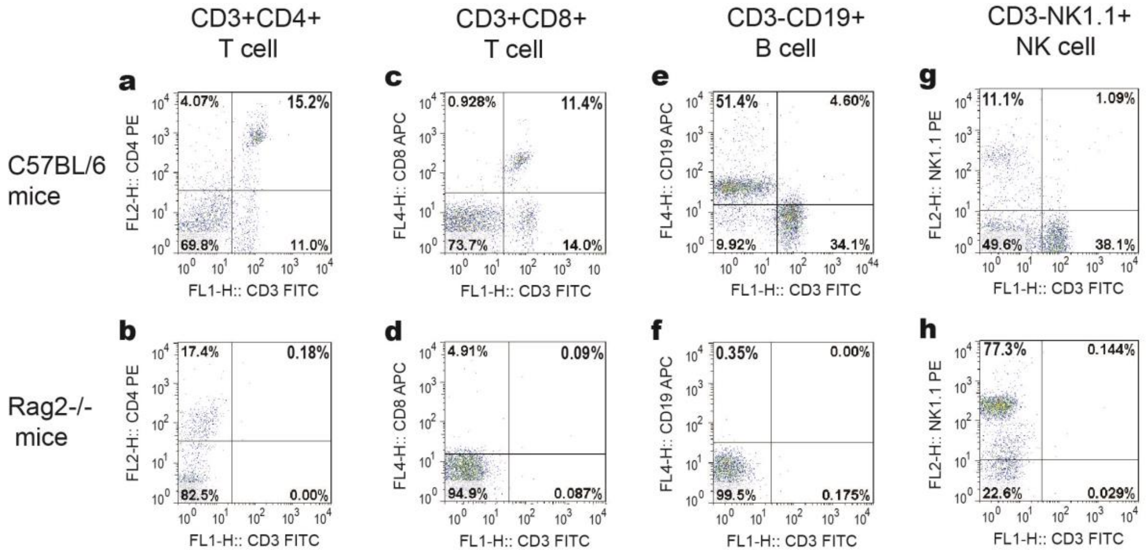

Supplementary Figure S1: Analysis of peripheral white blood cells in C57BL/6 and *Rag2*$^{-/-}$ mice. The presence of various lymphocyte populations in the peripheral blood of C57BL/6 and *Rag2*$^{-/-}$ mice was examined with flow cytometry. (a–d) The T cell compartment was analysed by simultaneous staining with either anti-CD3–fluorescein isothiocyanate (FITC; cat #553061) and anti-CD4–phycoerythrin (PE; cat #553652) (a, b) or anti-CD3–FITC and anti-CD8–allophycocyanin (APC; cat #553035) (c, d). (e–f) The B cell compartment was identified as containing cells that were negative for staining by anti-CD3–FITC and positive for staining by anti-CD19–APC (cat #550992). (g–h) The NK cell compartment was detected by double staining with anti-CD3–FITC and anti-NK1.1–PE (cat #557391). NK cells were identified as cells that were negative for staining by anti-CD3–FITC and positive for staining by anti-NK1.1–PE.

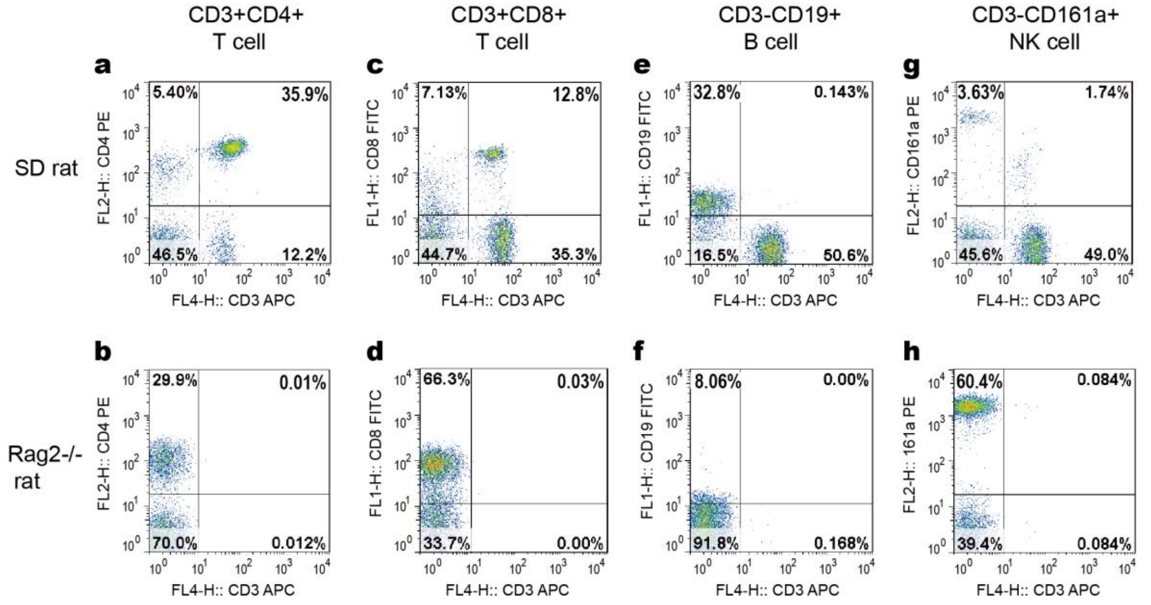

Supplementary Figure S2: Analysis of peripheral white blood cell populations in wildtype SD and immunodeficient *Rag2*$^{-/-}$ rats. The presence of various lymphocyte populations in the peripheral blood of SD and *Rag2*$^{-/-}$ rats was examined by performing flow cytometry. (a–d) The T cell compartment was analysed by simultaneously staining the cells with either with anti-CD3–allophycocyanin (APC; cat #557030) and anti-CD4–phycoerythrin (PE; cat #551397) (a, b) or anti-CD3–APC and anti-CD8–fluorescein isothiocyanate (FITC; cat #561965) antibodies (c, d). (e–f) The B cell compartment was identified as containing cells that were negative for staining by anti-CD3–APC and positive for staining by anti-CD19–FITC (cat #557398). (g–h) Peripheral blood cells were confirmed by double staining with anti-CD3–APC and anti-CD161a–PE (cat #555009) antibodies. The NK cells were identified as the cells that were negative for staining by anti-CD3–APC and positive for staining by anti-CD161a–PE.
